# Relationship of skin complexion with gingival tissue color and hyperpigmentation. A multi-ethnic comparative study

**DOI:** 10.1186/s12903-024-04189-7

**Published:** 2024-04-13

**Authors:** Pradeep Koppolu, Haifa Almutairi, Safa al Yousef, Nisren Ansary, Mohammed Noushad, Mantri Bharath Vishal, Lingam Amara Swapna, Nouf Alsuwayyigh, Malak Albalawi, Deepti Shrivastava, Kumar Chandan Srivastava

**Affiliations:** 1 UWA Dental School, The University of Western Australia, Perth, Australia; 2 Department of Preventive Dental Sciences, College of Dentistry, Dar Al Uloom University, Riyadh, Saudi Arabia; 3 Department of Restorative and Prosthetic Dental Sciences, College of Dentistry, Dar Al Uloom University, Riyadh, Saudi Arabia; 4 U&V Dental Clinic Implant & Laser Centre, Hyderabad, India; 5 Department of Surgical and Diagnostic Sciences, College of Dentistry, Dar Al Uloom University, Riyadh, KSA Saudi Arabia; 6 Department Department of Preventive Dentistry, College of Dentistry, Jouf University, Sakaka, 72345 Saudi Arabia; 7 Department of Oral & Maxillofacial Surgery & Diagnostic Sciences, College of Dentistry, Jouf University, Sakaka, 72345 Saudi Arabia; 8 Department of Oral Medicine & Radiology, Saveetha Institute of Medical and Technical Sciences, Saveetha Dental College, Saveetha University, Chennai, Tamil Nadu India

**Keywords:** Complexion, Gingival pigmentation, Skin color, Melanin

## Abstract

**Background and Objective:**

The most frequently seen intra-oral soft tissue is the gingiva. Most often, it is seen as coral-pink tissue that surrounds the neck of the teeth. Gingiva that encircles the tooth necks and covers the alveolar processes of the jaws is an intra-oral tissue that exhibits biomimetic features. The wide range of colors of the gingiva depends on the configuration of gingival vascularity, the degree of epithelial cornification, level of melanogenesis, and the depth of epithelialization. However, the color of the gingiva varies depending on the degree of melanin pigmentation. The current study aimed to identify the different distribution patterns of gingival color and determine the correlation between skin color, gender, and geographical area of origin.

**Materials and methods:**

A total of 839 subjects were involved in the study where the gingival color and skin tone were measured using the Dummett-Gupta Oral pigmentation Index (DOPI) combined with VITA VMK MASTER and skin shade method developed by Revlon (USA) and L’Oreal (France) for makeup foundation shades. One investigator was calibrated for the examination of the colors after being tested for normal color vision and color aptitude using the line test.

**Results:**

A significant association was found between skin color and gingival pigmentation (χ2 value (6) = 114.48; *P* = 0.001). It was also found that females (67.1%) significantly had darker gingiva than males (58.3%). The study statistics display that location of the individual was also statistically associated with melanin pigmentation of the gingiva (χ2 value (57) = 559.33; *P* = 0.001).

**Conclusion:**

The study concluded that gender, skin color, and individual location are significantly associated with gingival melanin pigmentation.

## Introduction

The gingiva is the region of the oral mucosa that encircles the tooth necks and covers the alveolar processes of the jaws. The “coral pink” color of the gingiva is a result of the vascular supply, the thickness and degree of keratinization of the epithelium, and the existence of cells that contain pigment [1].

The gingiva is the intra-oral tissue that is most frequently colored and most easily seen. Healthy gingiva can range in color from pale pink to bluish-purple. A wide range of colors exist, and they depend on the level of melanogenesis, the degree of epithelial cornification, the depth of epithelialization, and the configuration of gingival vascularity [2]. 

Black people’s oral pigmentation is distributed as follows: tongue 15%, mucous membrane 22%, gingiva 60% and hard palate 61% [3]. 

The primary pigment that gives tissues their color is melanin. It can start to show up in the oral tissues as early as three hours after birth and, in some cases, is the only indication of pigmentation in the body. The cells called melanocytes, which are dendritic cells of neuroectodermal origin found in the basal and spinous layers of the gingival epithelium, produce this non-haemoglobin-derived pigment [4]. 

Although gingival melanin hyperpigmentation seldom causes medical issues, some people may find their dark gums unsightly. Patients with an “excessive gingival display” are more likely to experience this issue [5]. The removal or reduction of gingival hyperpigmentation using a variety of procedures is known as gingival depigmentation [6]. The primary justification for depigmentation therapy is a person’s desire for better aesthetics. Better treatment plans can be developed by having a better understanding of the pigmentation distribution.

The studies by Sepolia et al. [5] and Deepak et al., [6] have important implications in the field of dentistry, particularly around biomimetic esthetic dentistry. Sepolia et al. [5]., fills a literature gap by providing a detailed analysis of the amount of gingival visibility during natural and forced smiles in patients. The study provides clinicians with a classification system that can be used to determine the amount of gingival visibility in patients and can aid in the development of biomimetic treatment plans for patients with excessive gingival display. On the other hand, Deepak et al., [6]. , discusses the importance of gingival health and appearance in the harmony of a biomimetic smile. The study provides clinicians with various biomimetic treatment modalities for depigmentation of gingiva, which can help in addressing common patient complaints of ‘black gums’, particularly in patients with a very high smile line. The study highlights the importance of considering gingival tissues in the development of a biomimetic attractive smile and the need to address gingival pigmentation issues to achieve optimal biomimetic esthetic outcomes. The findings of both studies have implications for the management of patients seeking biomimetic esthetic dental treatments. Understanding the amount of gingival visibility and addressing issues of biomimetic gingival pigmentation can lead to improved patient satisfaction and can enhance the overall biomimetic esthetic outcome of dental treatments. Clinicians can use the classification system provided by Sepolia et al., [5]. , to develop individualized biomimetic treatment plans for patients with excessive gingival display. Meanwhile, the biomimetic treatment modalities discussed by Deepak et al., [6]. , can serve as a guide for clinicians in the biomimetic management of gingival pigmentation issues

The primary need for this biomimetic study was to investigate the association between gingival color and participants geographical area of origin. It was assessed that previous studies have not explored this biomimetic relationship or have not examined it in detail across a wide range of nationalities as far as the literature on this topic is concerned. Therefore, this biomimetic study could potentially contribute to filling a gap in the literature regarding the relationship between gingival color and participants geographical area of origin [7]. Additionally, the findings of this study could inform future research exploring potential underlying factors that may explain the observed differences in gingival color across nationalities.

The current study aimed to identify the different distribution patterns of gingival color and determine the correlation between skin color, gender, and participants geographical area of origin. The null hypothesis was that there is no association of skin complexion with gingival color and pigmentation.

## Materials and methods

### Study characteristics

The ethical committee of the institute COD/2020/111 acceded to the study. The present study is an observational, cross-sectional study.

### Sample characteristics

A total of 839 subjects in the age group of 18–35 years were included in the study of which 464 were males and 375 were females who were attending the dental clinics at the university. The mean age of females is 23.2 ± 5.3 and males is 28.3 ± 6.7. The sample size can be determined using the following formula:

$$n^\circ \, = \,Z_{_{1 - \alpha /2^\circ }}^2\, \times \,(1\, - \,P)/{ \in ^{2^\circ }}\, \times \,P$$

The computed sample size was 801, rounded to 838, considering the anticipated percentage of 66.7% from the pilot study, with a relative precision of 5% and a desired confidence level of 95%.

The objectives and the procedures of the study were properly explained to the patients, after which an informed consent was acquired from all the subjects. Subjects with non-mottled and uniformly pigmented gingiva, distinctly light, medium, and dark skin color were included in the study. Patients with periodontitis or any gingival pathology including drug or chemical pigmentation, color changes, mottling and who were smokers were excluded from the study. Subjects with chemical skin toning, albinism were also excluded from the study.

### Study protocol

**Fig. 1:**
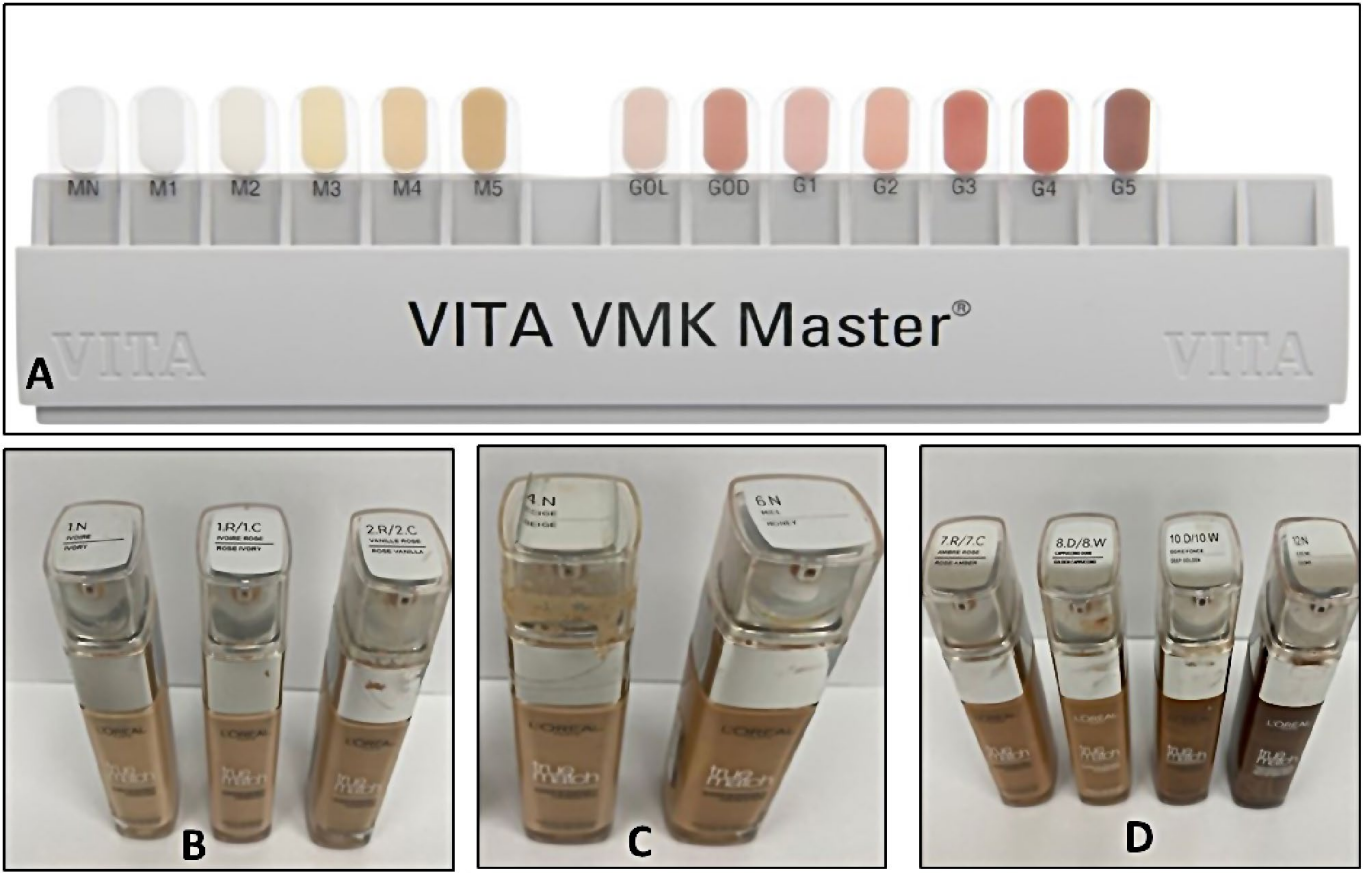
**(A-D)**: **A**- VITA VMK MASTER Gingival Shade Guide; **B** - Light Skin Shade; **C** - Medium Skin shade; **D** - Dark Skin Shade

The assessment was conducted in the same examination room with the same lighting conditions by a trustworthy, calibrated examiner who passed the Ishihara test for color blindness. The participants were set an arm’s length distance from the examiner and the face of each member was set upright so that the mouth was at the level of the clinician’s eyes. The gingival color was graded using the Dummett-Gupta Oral pigmentation Index (DOPI) combined with VITA VMK MASTER (Fig. 1A) [2] The criteria are as follows:

- 0 = Pink tissue (No clinical pigmentation) [VITA GOL].
- 1 = Mild, light brown tissue (Mild clinical pigmentation) [VITA G1, G2].
- 2 = Medium brown or mixed pink or brown tissue (Moderate clinical pigmentation) [VITA G3, G4, GOD].
- 3 = Deep brown or blue/black tissue (Heavy clinical pigmentation) [VITA G5].

Investigators assessed color aptitude and normal color vision using the line test, and a comparison of observers and light sources was done with a color rule. A rapid selection of shade in daylight was used to avoid fatigue on the examiner corneas. Later examining the colors using the skin shade method made by L’Oreal (France) for the purpose of makeup foundation shades selection, skin shade was measured twice on the inner aspect of the wrist of participants, (Fig. 1B-D) [8]. The obtained data was subject to statistical analysis.

### Statistical analysis

The descriptive statistics were expressed as a frequency and percentage. Chi-square analysis was used to calculate the percentage change in categorical variables.

## Results

**Fig. 2:**
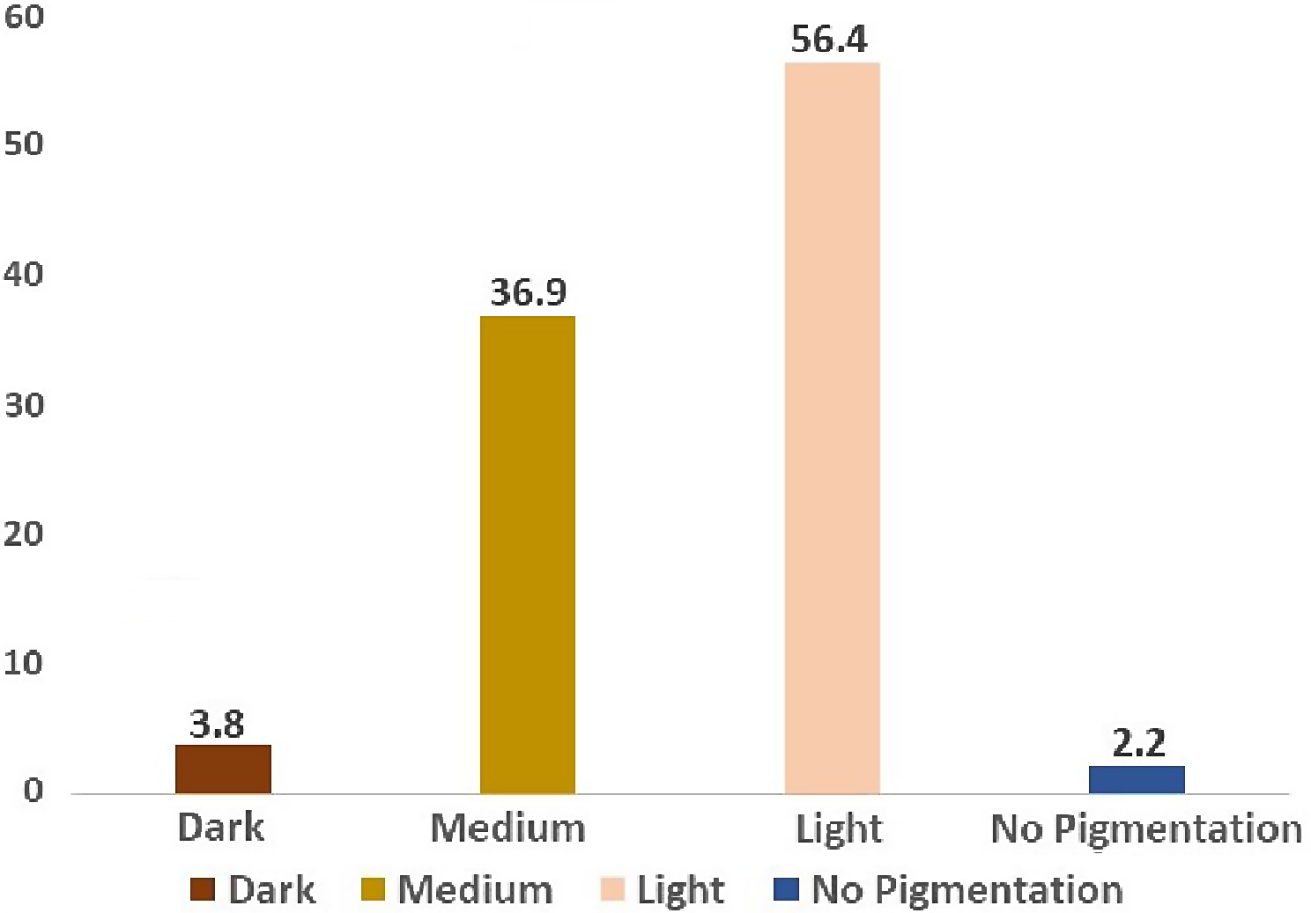
Graph showing the gingival category being distributed among the study population

**Fig. 3:**
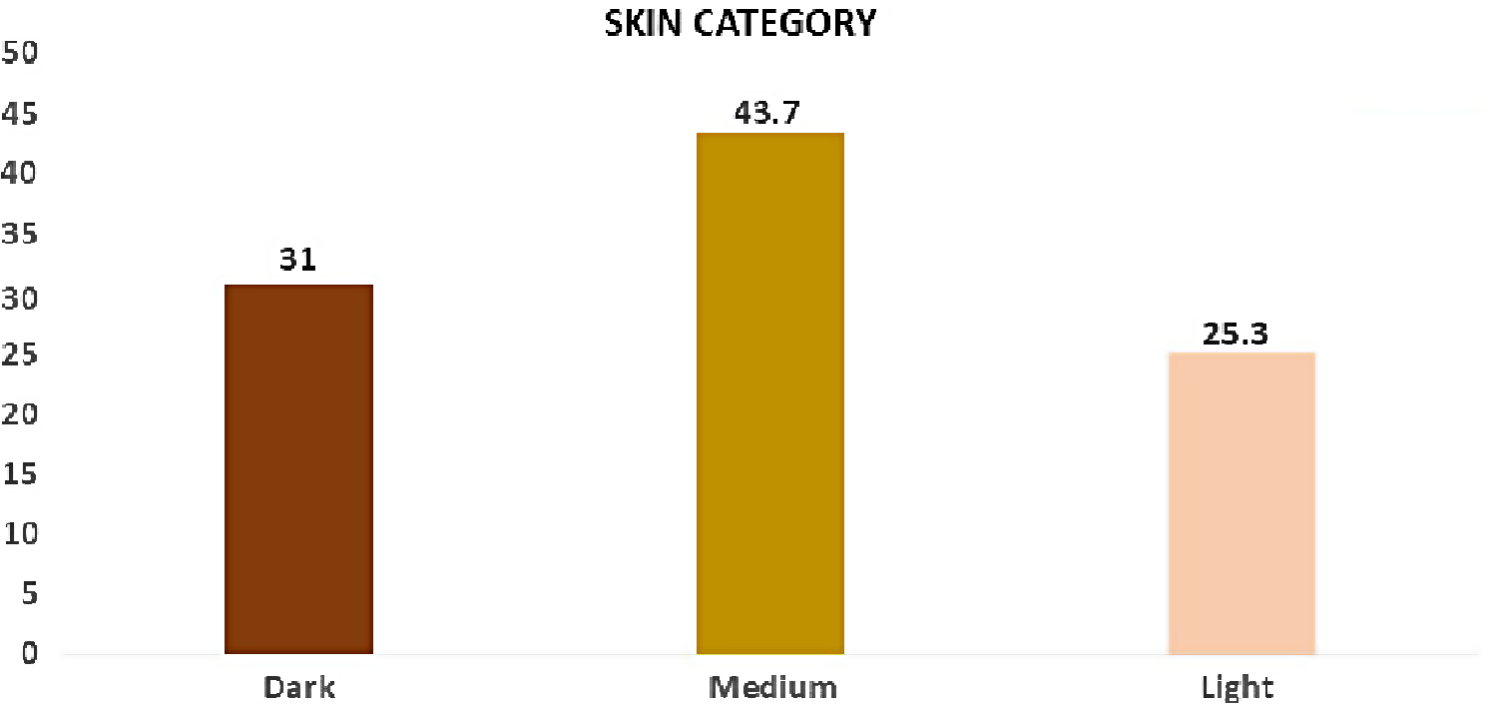
Graph showing the skin category being distributed among the study population

Our research study comprised of 839 patients. Out of which 464 were males and 375 were females’ participants, respectively. Based on a thorough clinical intraoral examination, the severity and location of gingival pigmentation; four categories were defined light, medium, dark and no pigmentation Since the study was conducted in Saudi Arabia most of the participants belonged to Saudi nationality. A categorization scheme outlining the patterns of anatomic distribution of gingival pigmentation was developed based on the examination of the individuals. The distribution of the gingival category and skin category among the study population was represented in Figs. 2 and 3. For men, the highest category of gingival pigmentation is medium (51%) and for women it is 64.2%.

**Fig. 4:**
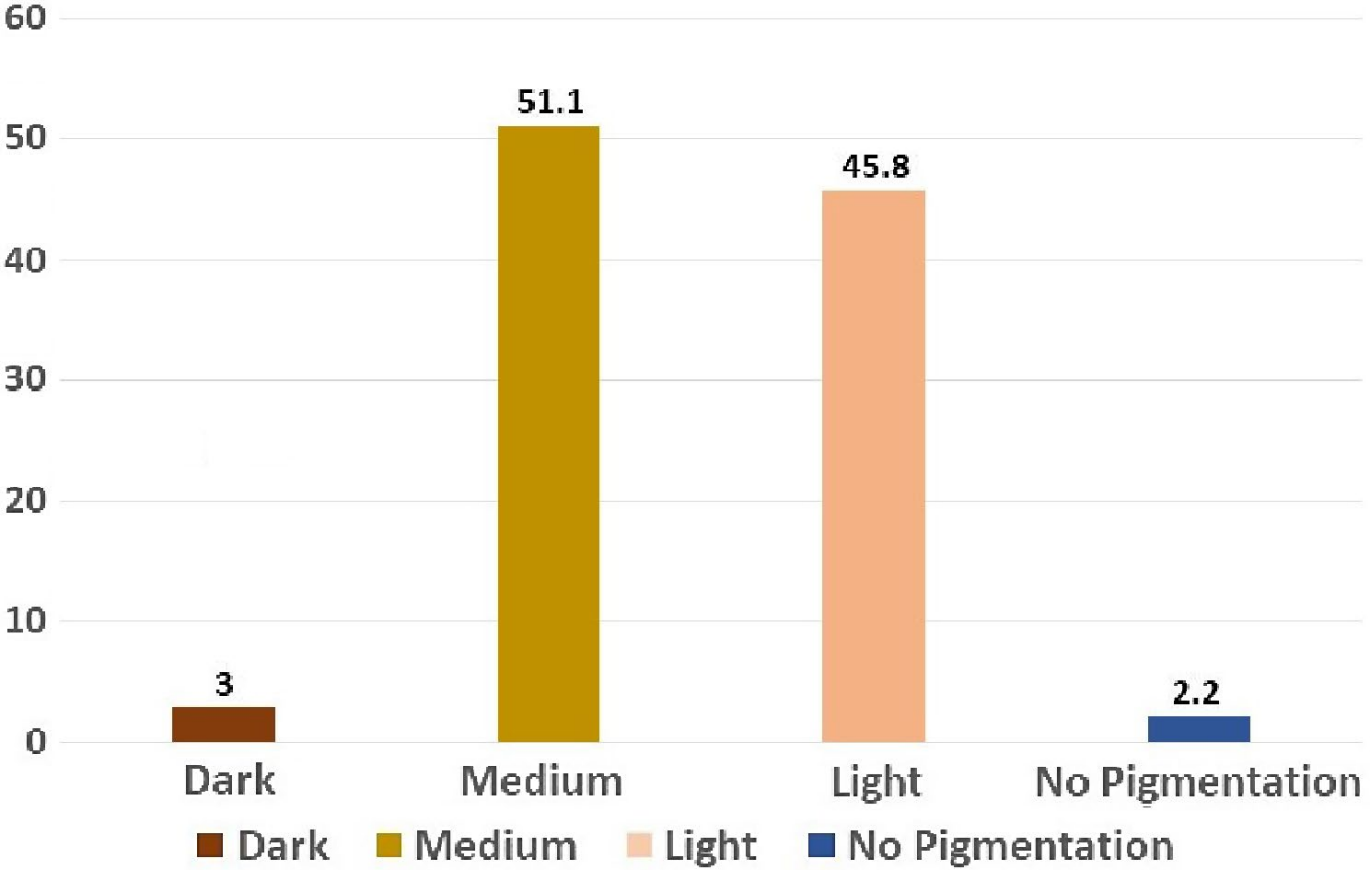
Percentage distribution of gingival pigmentation among males

**Fig. 5:**
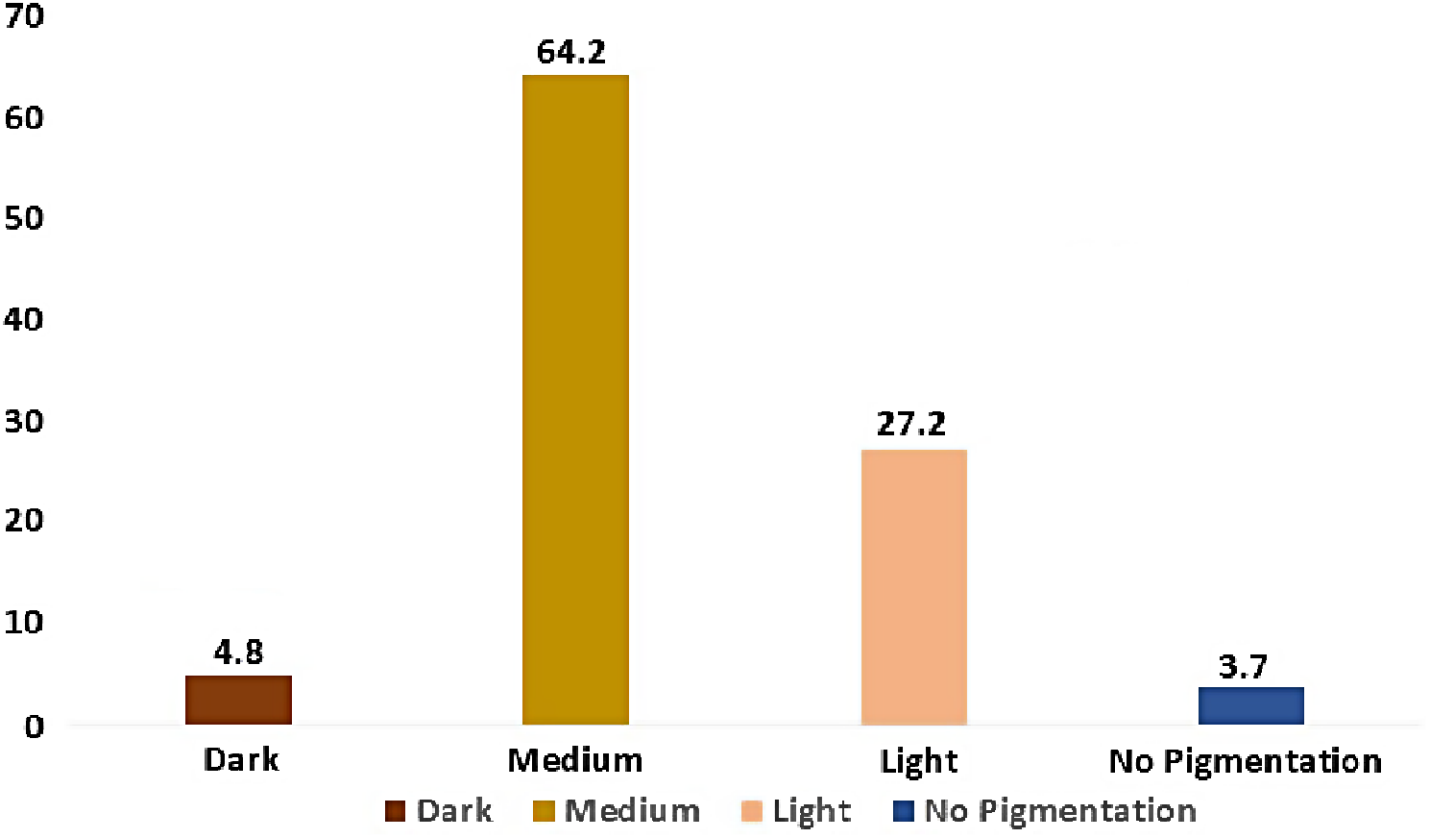
Percentage distribution of pigmentation among females

Among non-pigmentation category 41.7% were males and 58.3% were females, among light pigmentation category 67.10% were males and 32.90% were females. The medium gingival pigmentation category had many participants with 49% being males and 51% being females. The dark pigmentation category males were 43.8% and females being 56.2%. Among the male participants majority (232) 50% of them had medium pigmentation, same is the observation in female participants with 64.2% (241). Among male participants 3% (14) had dark pigmentation, 45.8% (208) had light pigmentation and 2.2% (10) had no pigmentation. Among female participants 4.8% (18) had dark pigmentation, 27.2% (102) had light pigmentation and 3.7% (14) did not have any pigmentation respectively **(**Figs. 4 and 5**)**. The association is statistically significant (χ2 value (3) = 28.46; *P* = 0.001) (Table 1).

**Table 1:** Association of the gingival color category with gender

Gender	Gingival Color Category				df	χ2 value	P value
	Dark	Medium	Light	No Pigmentation			
Male	14 (43.80)	232 (49)	208 (67.10)	10 (41.70)	3	28.462	0.001**
Female	18 (56.20)	241 (51)	102 (32.90)	14 (58.30)			

*Note* Results are expressed in number (%)

**P value < 0.01; df: degrees of freedom; χ2 value: Chi square variate

The study statistics display that most subjects from India (90.28%), Egyptian (67.44%), Sudan (66%), Philippines (65.7%), Nepal (63.16%), Yemen (63%) and Saudi (61.4%) were associated with a medium gingival category. In contrast, most subjects from Afghanistan (100%), Palestinian (100%) and Somalia (83%) belongs to Light gingival category. The majority of Uganda (71.4%) and Srilanka (37.5%) subjects belong to the Dark Gingival category. The majority of Ethiopia (40%), Nepal (31.58%), and Syria (8.16%) subjects belong to the No pigmentation gingival category. And the association is statistically significant. (χ2 value (57) = 559.33; *P* = 0.001). **(**Table 2**)**

**Table 2:** Association of the gingival color categories with nationality

Nationality	Gingival Color Category				df	χ2 value	P value
	Dark	Light	Medium	No pigmentation			
Afghani	0	20 (100)	0	0	57	559.33	0.001**
Bangladesh	4 (12.50)	10 (31.20)	18 (56.25)	0			
Egyptian	3 (6.90)	11 (25.58)	29 (67.44)	0			
Ethiopian	0	9 (45)	3 (15)	8 (40)			
Indian	0	7 (9.72)	65 (90.28)	0			
Indonesian	0	11 (42.30)	15 (57.70)	0			
Jordan	0	17 (44.70)	21 (55.30)	0			
Kenyan	0	16 (50)	16 (50)	0			
Moroccan	0	9 (45)	11 (55)	0			
Nepal	0	1(5.20)	12 (63.16)	6 (31.58)			
Pakistan	0	15 (47)	17 (53)	0			
Palestinian	0	18 (100)	0	0			
Philippines	0	12 (34.30)	23 (65.70)	0			
Saudi	6 (2.00)	101 (34.50)	180 (61.40)	6 (2.00)			
Somali	0	10 (83)	2 (17)	0			
Srilanka	6 (37.50)	2 (12.50)	8 (50)	0			
Sudan	3 (17)	3 (17)	12 (66)	0			
Syrian	0	26 (53)	19 (38.77)	4 (8.16)			
Uganda	10 (71.40)	1 (7.14)	3 (21.40)	0			
Yemen	0	11 (37)	19 (63)	0			

*Note* Results are expressed in number (%)

**P value < 0.01; df: degrees of freedom; χ2 value: Chi square variate

According to the Chi-square test, most medium skin groups (31.7%) relate to the Medium gingival category, whereas most Dark skin (90.6%) patients are associated with the Dark gingival category. Most persons with light skin (61.9%) fall into the Light Gingival group. And the association is statistically significant. (χ2 value (6) = 114.48; *P* = 0.001) (Table 3).

**Table 3:** Association of the gingival color category with Skin Category

Skin Category	Gingival Color Category				df	χ2 value	P value
	Dark	Light	Medium	No pigmentation			
Dark	29 (90.60)	63 (20.30)	159 (33.60)	9 (37.50)	6	114.486	0.001**
Light	0	192 (61.90)	164 (34.70)	11 (45.80)			
Medium	3 (9.40)	55 (17.70)	150 (31.70)	4 (16.70)			

*Note* Results are expressed in number (%)

**P value < 0.01; df: degrees of freedom; χ2 value: Chi square variate

## Discussion

The pigment melanin, produced naturally by our bodies, induces pigmentation on the gingiva and the skin. This usually results in “Black Gingiva,” where appearance is critical, particularly if the patient has an “Excessive gingival display.” Gingival depigmentation is performed to address this issue, raise the patient’s cosmetic value, and remove the hyperpigmentation of the gingiva [9]. The overall prevalence of melanin pigmentation in various groups has been observed to range from 0 to 89% [10]. There is considerable variation in the degree of melanin pigmentation between different ethnicities/races and between individuals within the same ethnicity/race, and these variations are normal. Genetic, metabolic, endocrine, chemical or physical factors and environmental factors are involved in non-physiological changes in melanin pigmentation in the oral mucosa. The most widely used system is the Munsell system, which is also among the best suited systems for soft tissue color assessment because of its large color palette. In this study, natural light was used for visual assessment. Traditionally, natural gingival color has been studied visual comparison methods using the Munsell color system, it is reported gingival color varies with the position of papillary, attached, and marginal gingiva. According to Manson J. D., Eley B. M, and de Kromet C. J. et al., healthy gingiva in Caucasians ranges in hue from pale pink to coral pink. At the same time, in African Americans or Asian-Americans, the rear portions are brown to blue [11, 12]. Among the various ethnic groups, Indians have a wide variation in their skin color. Therefore, from fair to dark, there are all types of skin tones distributed in the whole country [13]. It showed a significant relationship between skin color and gingival pigmentation. In 85% of cases, the color of extra-oral tissue (such as the cheeks) is a good indicator of the gingiva and mucosa color. Several studies showed that subjects with fair skin had minor gingival pigmentation, whereas those with dark skin had severe pigmentation, and agreed with the current study [9, 14, 15].

The study found that individuals with darker skin had higher levels of gingival melanin pigmentation compared to those with light skin individuals. Since our study excluded the smokers the variability in the pigmentation could be attributed majorly to genetics and melanoblastic activity. Individuals with dark skin tend to have higher levels of eumelanin, which is more photoprotective. Physiologic pigmentation can manifest as multifocal or diffuse melanin pigmentation, with varying incidence among ethnic groups. In dentistry, aesthetics refers to both whiter teeth and pink gingiva, which is always a challenging undertaking for the treating periodontist.

In the current study, it was found that the people of Saudi Arabia had the greatest gingival pigmentation, and the least was found among the Syrians, Nepalis, and Ethiopians. Tamizi et al. [16] theorized that eumelanin-producing melanocytes were present in comparatively high quantities in the earliest modern humans. They matched the native inhabitants of Africa today who have a darker complexion and found that melanocytes were more numerous in them. Some of these individuals moved and lived in regions of Asia and Europe, where the eumelanin production diminished in temperatures with less intense solar exposure, resulting in less skin and oral pigmentation. Consequently, individuals of African, East Asian, or Hispanic ancestry typically exhibit significant levels of oral melanin pigmentation [17].

In the current study, a strong association between pigmentation and gender was found. Females presented with greater gingival pigmentation when compared to males. However, studies conducted by Kaur H et al., and Gorsky M et al. found no correlation between pigmentation and sex. Similarly, the results of Steigman and Reade, who studied a group of Australian Aborigines, showed that there was no significant correlation between pigmentation of gingiva and gender [16–21]. Environmental or genetic variation could be the source of the color differences between men and women. Our study is also in agreement with Verma et al. who stated that the skin tone and gingival pigmentation were observed to correlate and observed that the darker the skin tone, darker is the gingival pigmentation [22] significantly positively. Our study agrees with Dosumu and Dosumu a study done in 2010 and Ponnaiyan et al. showed a significant correlation between facial skin and gingival pigmentation [9, 23].

In the current study, the Dummett-Gupta Oral Pigmentation Index (DOPI) combined with VITA VMK MASTER was used to evaluate the color tone of the gingiva. This index represents the assignment of a composite numerical value to the total melanin pigmentation manifested on clinical examination of various oral tissues. Visual evaluation can be influenced by the observer’s experience, the quality of the light source, the type of hue, and the match of the evaluator’s eye color. Our assessments of gingival pigmentation and skin color are subjective and is lacking in external validity in some way. Due to the shortcomings in the study protocols that were employed in the current study, care should be taken when interpreting the study results.

The skin color was measured using the skin shade method developed by Revlon (USA) and L’Oreal (France) for makeup foundation shades, even though the methods employed in this study to evaluate skin tones were not quantifiable. The methods used to assess gingival appearance might have been subjective; they could be used as a standard to measure the color against and could serve various guidelines.

### Limitations of the study and future directions

The limitations of this study include the sample was limited to individuals attending a single dental school in Saudi Arabia, which may have introduced selection bias and limited the generalizability of the findings to other populations. Additionally, the use of self-reported nationality may have also introduced inaccuracies in the data, as individuals may identify with different ethnicities and cultures beyond their country of origin. The number of subjects among all ethnic groups also varied that could have influenced the result of the analysis. Further studies utilizing longitudinal or experimental designs are needed to determine the directionality and nature of this relationship.

## Conclusion

In the current study, it was found that there was a strong association between skin tone and pigmentation. Gender and location were also found to be significantly associated with gingival pigmentation. Through this study, researchers can acquire valuable knowledge regarding the variations in gingival color and comprehend potential correlations with individual attributes. This study has the potential to enhance comprehension of oral health and furnish vital insights for dental practitioners and researchers in the sector. The findings of this study can also be used to inform patients and choose the most suitable surgical approach for gingival depigmentation. Patients, when provided with knowledge regarding various surgical methodologies, their advantages, hazards, and potential results, can actively engage in decision-making.

## Data Availability

The data will be available on reasonable request from the corresponding author. The data are not publicly available due to concerns pertaining to privacy and identity of the consenting participants that were involved in this study.
